# Profile of candidiasis in HIV infected patients

**Published:** 2012-12

**Authors:** Khan P Anwar, A Malik, Khan H Subhan

**Affiliations:** 1 Department of Microbiology J.N.M.C.H, Aligarh Muslim University, Aligarh; 2 Department of Internal Medicine J.N.M.C.H, Aligarh Muslim University, Aligarh

**Keywords:** HIV, AIDS, Candida, CD4 count

## Abstract

**Background and Objectives:**

Candidiasis is a common opportunistic infection in HIV-infected patients. The spectrum of Candida infection is diverse, starting from asymptomatic colonization to pathogenicforms. The low absolute CD4+ T-lymphocyte count has traditionally been cited as the greatest risk factor for the development of Oropharyngeal Candidiasis and current guidelines suggest increased risk once CD4+ T lymphocyte counts fall below 200 cells/µL. Gradual emergence of non-*albicans Candida* species as a cause of refractory mucosal and invasive Candidiasis, particularly in patients with advanced immunosuppression and problem of resistance to azoles and other antifungal agents in the Candida species is a point of concern.

**Materials and Methods:**

A prospective study was carried out over a period of 2 years (2010-2011) on patients suffering from AIDS for the presence of candida infection. After thorough clinical examination relevant specimens were collected and processed specifically to ascertain candida infection. Speciation of candida isolates and antifungal sensitivity testing was also done. The CD4 cell counts of all the patients were estimated and correlated with the presence (or absence) of candidiasis.

**Results:**

Out of a total of 165 HIV positive patients, a definitive diagnosis of candidiasis was made in 80 patients. *Candida albicans* was the most common yeast isolated. Patients with candidiasis had CD4 counts less than 200 cells/mm^3^. Maximum resistance was seen with fluconazole while no resistance was seen with voriconazole.

**Conclusion:**

The most common opportunistic fungal infection in HIV positive patients is candidiasis, affecting the mucocutaneous system mainly but the invasive form is also common. Resistance to azoles and other antifungal agents in the Candida species is a point of concern.

## INTRODUCTION

Risk of many HIV-related diseases varies with the patient's degree of immunosuppression. CD4 counts and quantitative HIV-1 RNA levels are most commonly used as surrogate markers of immune function (1, 2).Although the introduction of antiretroviral therapy (ART) has had a major impact on theinfectious complications of AIDS (3), Candidiasis still remains a common opportunistic infectionin HIV-infected patients (4, 5, 6).

The spectrum of Candida infection is diverse, starting from asymptomatic Colonization to Oropharyngeal Candidiasis (OPC), esophagitis,onychomycosis, vulovaginitis, cutaneous Candidiasis and systemic candidiasis or invasive candidiasis including candedemia (7, 8, 9). Oropharyngeal candidiasis,often the first sign of HIV infection, is the most prevalent fungal opportunistic infection inHIV-infected individuals (10). Prior to the availability of active antiretroviral therapy, oropharyngeal Candidiasis was a very common finding in patients with HIV/AIDS. With the development of effective anti-retroviraldrugs, rates of oral Candidiasis are reported to decrease (2, 5, 6).

The low absolute CD4+ T-lymphocyte count has traditionally been cited as the greatest risk factor for the development of OPC and current guidelines suggest increased risk once CD4+ Tlymphocyte counts fall below 200 cells/µL. OPC remains more common in HIV infected patients than those with a similar degree of immunosuppression (bone marrow transplant or patients receiving chemotherapy). This observation suggests that HIV itself may play a role in host susceptibility (8). The defect in cellular immunity characteristic of HIV infection predisposes to Candida infections. Role of lymphocytes and polymorphonuclear leukocytes in defense against infection with Candida is of great importance, as indicated by the relatively common occurrence of disseminated candidiasis in neutropenic patients without defects in cellular immunity (9).

This study was conducted in HIV positive patients to find out the prevalence of Candidiasis and various Candida species implicated, and to study the relationship of Candida infections to immunological markers represented by CD4 T+ cells and Absolute Lymphocyte counts.

## MATERIAL AND METHODS

A prospective study was carried out over a period of2 years (2010-2011) on patients suffering from AIDS for the presence of Candida infection.

The HIV status of all the patients was confirmed at ICTC, J.N. Medical College and Hospital. The HIV antibody status was assessed by three ERS (ELISA, Rapid, and Simple) tests as recommended by the National AIDS Control Organization (NACO), Ministry of Health and Family Welfare, Government of India (Guidelines for HIV testing, March 2007).

Various clinical specimens including blood, oral swabs, expectorated or induced sputum, skin/nail scrapping and other specimens were collected according to the patient's clinical presentation. All samples were collected under complete aseptic conditions and transported immediately to the microbiology laboratory and processed specifically to ascertain candida infection.

Blood sample for fungal culture was collected by venipuncture and inoculated in two biphasic blood culture bottles (BHI agar and broth), one each being kept at 25°C and 37°C, respectively. Swabs and other specimens were inoculated onto Sabouraud dextrose agar tubes containing chloramphenicol. Culture tubes incubated and were observed daily for the growth. The Candida isolatesthus obtained were identified and characterized on the basis of colony characters, germ tube production, morphology on corn meal agar, sugar fermentation tests, sugar assimilation tests, and the color of the colony on CHROMagar (11).

Antifungal susceptibility testing for all candida isolates was performed by the broth microdilutionmethod as per Clinical and laboratory Standard Institute (CLSI). Interpretative guidelines for susceptibility testing in vitro for yeasts are given in Table 1. Standard antifungal powders of fluconazole, ketoconazole, itraconazole (HiMedia. India), and voriconazole (Pfizer, New York, USA) were obtained from the respective manufacturers. The patients in whom esophageal Candidiasis was suspected underwent oesophagoscopy by a clinical expert. The CD4 cell counts of all the patients were estimated by Flow-Cytometry using Partec CyFlow Counter (Germany).

**Table 1:** Interpretative guidelines for susceptibility testing in vitro for yeasts.

Antifungal agents	Susceptible (S) MIC (µg/ml)	Susceptible dosedependent (S-DD) MIC (µg/ml)	Resistant (R) MIC (µg/ml)
**Fluconazole**	≤ 8	16–32	≥ 64
**Ketoconazole**	≤ 0.0625	0.125–0.5	≥1
**Itraconazole**	≤ 0.125	0.25–0.5	≥ 1
**Voriconazole**	≤ 0.5	1–2	≥ 4

## RESULTS

Out of a total 165 HIV positive patients a definitive diagnosis of candidiasis was made in 80 patients. Various presentations of candidiasis in HIV positive patients include doropharyngeal candidiasis, oesophageal candidiasis, candidemia, pulmonary candidiasis, cutaneous candidiasis and candidal diarrhea (Table 2). Most common presentation was oral candidiasis, seen in 57(71.25%) patients out of them 5(6.25%) patients had also had oesophageal candidiasis. Two patients had candidemia (2.5%) and 1 had pulmonary candidiasis with candedemia, this patient had the lowest CD4 count (36 cells/mm^3^) and total lymphocyte count (582 cells /mm^3^) among all the patients with candidiasis. Cutaneous candidiasis was present in 9 (11.25%) patients and 4 (5%) patients had candida diarrhea.

**Table 2:** Distribution of Various presentation of Candidiasis and CD4 counts in HIV positive patients.

Disease	No. of Patients (n = 80)	Mean CD4 (± SD)	CD4 Range (cells/mm^3^)	Mean Total lymphocyte count (cells/mm^3^)
**Oral Candidiasis only**	52	147.8 (±77.0)	22-350	858(±231.0)
**OralCandidiasis+ Oesophageal Candidiasis**	05	134.2 (±90.5)	46-267	762(±153.0)
**Candidemia**	02	112.5 (±1.5)	111-114	689(±47.0)
**Candedemia + Pulmonary Candidiasis**	01	36	–	582
**Candida diarrhea**	04	126.7 (±63.8)	52-235	753(±324.0)
**Cutaneous Candidiasis**	9	181.8 (±99.7)	33-296	906(±353.0)

Demographic characteristics of the 165 HIV-infected individuals with and without candidiasis are described in Table 3. The two groups were almost similar in regard toage, gender and the mode of transmission of HIV. With reference to the immune status, 62 (77.5%) patients with Candidiasis had CD4 counts less than 200 cells/mm^3^and 18 (22.5%) had a CD4 count of more than 200cells/mm^3^, while candidiasis 26 (30.56%) candidiasis had CD 4 counts less than 200 cells/mm^3^ and 59 (69.41%) had a CD4 count of more than 200cells/mm^3^. Total lymphocyte counts were significantly lower in patients with Candidiasis (868cells/mm^3^) as compared to patients without candidiasis (1760 cells/mm^3^).

**Table 3:** Demographic and clinical characteristics of patients with and without Candidiasis.

Characteristics	With Candidiasis (80)	Without Candidiasis (85)	p-value

Sex	No. of Patients (%)		
Male	44 (55)	48 (56.4)	–
Female	36 (45)	37 (43.5)	–
Age (mean)	32	29	–
Mode of HIV infection			
Sexual	60	67	–
Blood and products	7	5	–
Injection drug user	2	0	–
Not specified	11	13	–
CD4count			
<200 (cells/mm^3^)	62 (77.5)	26 (30.56)	<0.001
>200 (cells/mm^3^)	18 (22.5)	59 (69.41)	<0.05
Total lymphocyte count	868	1760	<0.01
Tuberculosis	36	24	–

Ninety four isolates of *Candida* specieswere retrieved from 80 cases of candidiasis of various organs, Fifty eight and 36 strains respectively of *Candida albicans* and Non-*albicans Candida* including *C. guilliermondi* (14 isolates), *C. parapsilosis* (9 isolates), *C. tropicalis* (7 isolates), *C. dubliniensis* (6 isolates) (Table 4). *Candida albicans* was the most common yeast isolated. Susceptibility of Candida isolates to various antifungal agents is shown in Table 5; maximum resistance was seen with fluconazole while the no resistance was seen with voriconazole.

**Table 4:** Distribution of various species of Candida isolated.

Candida species	Clinical specimens						Total

	Oral swab	Oesophageal biopsy	Blood	sputum	skin	stool	
**C. albicans**	41	2	1	2	7	5	58
**C. tropicalis**	04	0	2	0	0	1	07
**C. guilliermondi**	12	0	0	0	2	0	14
**C. dubliniensis**	05	0	0	1	0	0	06
**C.parapsilosis**	06	0	0	0	3	0	09
**Total**	68	2	3	3	12	6	94

**Table 5:** Susceptibility of candida species isolated to various antifungal agents.

Isolates/antifungal drug	Fluconazole			Ketoconazole			Itraconazole			Voriconazole		

	S	S-DD	R	S	S-DD	R	S	S-DD	R	S	S-DD	R
*C. albicans* (58)	38	14	6	39	14	5	41	13	4	52	6	0
*C. tropicalis*( 7)	4	1	2	3	3	1	5	1	1	5	2	0
*C. guilliermondi*(14)	11	2	1	12	1	1	12	1	1	13	1	0
*C. dubliniensis* ( 6)	3	1	2	2	2	2	3	2	1	5	1	0
*C. parapsilosis*(9)	5	3	1	5	3	1	6	3	0	7	2	0
Total (94)	61	21	12	61	23	10	67	20	7	82	12	0

(R) – Resistant; (S) – Sensitive; (S-DD) – Susceptible dose-dependent

The improvement in CD4 cell counts of patients with candidiasis who were on ART was maximum in the regimen of the combination of Zidovudine, Lamivudine and Nevirapine (Table 6).

**Table 6:** Improvement of CD4+ count in different ART Regimen.

Drugs included in the regimen	Baseline CD4+ cell count (mean)	CD4+ cell count at 6 months (mean)	CD4+ cell count at 12 months (mean)
Zidovudine + Lamivudine + Nevirapine	127	193	252
Stavudine + Lamivudine + Nevirapine	145	189	199
Zidovudine + Lamivudine + Efavirenz	132	196	243
Stavudine + Lamivudine + Efavirenz	116	185	212

## DISCUSSION

The first step in the development of a candida linfection is colonization of the mucocutaneous surfaces (12). HIV infection is not only associated with increased colonization rates but also with the development of overt disease. During the course of HIV infection, the rate of Candida infection is inversely related to the CD4 counts of the patient which in turn depends on the use of Anti-retroviral treatment. The present study analyzed the spectrum and the prevalence of Candida infection and its association with the immunological markers and Anti-retroviral treatment status.

The mean age of 32 years and 29 years in patients with and without candidiasis respectively, reflects only the gross demographic variable in terms of the age group mostly affected by the HIV epidemic across India (13), without any preponderance of development of candidiasis at any specific age and sex.

Sexual route of transmission was documented in 78% of patients while blood found sexual mode of ttransfusion was implicated in 1.7% of total patients studied. Heterosexual route of transmission remains the major route in India and it is also reported by NACO and other studies as the major route of transmission. M. Vajpayee *et al*. (14) reported heterosexual route of transmission in 59.8% of cases. SK Sharma *et al*. (15) found sexual mode of transmission in 41.5% of patients and Anupriyawadhwa *et al*. (16) reported it in 53.3% of patients.

In our study, oral candidiasis was found to be the most common (71.25%) opportunistic fungal infection. Various studies have reported similar prevalence of candidiasis. B.C. Pruthvi *et al*. (17) reported candidiasis in 71% of HIV positive patients, Nagalingeswaran K *et al*. (18)in 70%,A. Singh *et al*. (19) in 65% and Anupriyawadhwa *et al*. (16) found candidiasis in 50% of the HIV positive patients. Other studies reported candidiasis in 23 to 27% of HIV positive patients (14, 20, 21).

Non-*albican*s Candida as an agent of oral candidiasis in HIV/AIDS patients is documented (16, 19) In a study by Ismail H Sahand *et al*. (22) on distribution of candida isolates from oral swabs of HIV-infected individual similar results with *Candida albicans* isolated from 52% of patients and non-*albicans candida* from the rest. Anupriyawadhwa *et al*. (16) found 40% of all candida isolates to be non-*albican*.

*C. dubliniensisis* an opportunisticfungal pathogen originally associated with oral candidiasis in AIDS patients and now found to cause invasive infection, primarily in immune compromised patients. C. *dubliniensis* appears to be an opportunistic pathogen and normally is a minor component of the oral flora of humans, as opposed to *C. albicans*. Immunosuppression and the use of antimicrobial agents, including anti-fungal agents, apparently permits *C. dubliniensis* to increase in numbers heavily colonize the oropharynx, and eventually cause disease, most often oral candidiasis in both adults and children. Approximately 25% of HIV infected patients may be colonized with the yeast and *C. dubliniensis* has been isolated from the oral cavity of approximately 30% of patients with AIDS and oral candidiasis (23).

In our study 12.76% of the candida species were resistant to fluconazole, other studies have also reported increased fluconazole resistance in *Candida albicans* and other species of Candida (24). Problem of resistance to azoles and other antifungal agents in the candida species including C. dubliniensis is a point of concern as this species has been found to develop a stable in vitro resistance to fluconazole (25). It is known that cross-resistance exists between the various antifungal agents (26, 27), and should such a resistant strain be shared by a number of patients would leave a limited choice of medication which would be effective once they develop candidiasis. Fluconazole (or Azole) resistance is predominantly the consequence of previous exposure to fluconazole (or other azoles), particularly repeated and long-term exposure (28). This may be because long term and repeated use of antifungal drugs is often required in AIDS patients; they are more vulnerable to infection with resistant strains. Also, *C. albicans* resistance has been accompanied by a gradual emergence of non-*albicans Candida* species as a cause of refractory mucosal candidiasis, particularly in patients with advanced immunosuppression (29)

In conclusion, the most common opportunistic fungal infection in HIV positive patients is candidiasis, affecting the mucocutaneous system mainly but the invasive form is also common. Several Candida species are implicated in candidiasis. Although *C. albicans* remains the most common species responsible for candidiasis, disease due to newer species like C. *dubliniensis* are also increasing. Routine checks for opportunistic infections including oropharyngeal candidiasis is important and should be carried out because it helps to monitor disease progression and it prevents complications such as candidemia. Identifying Candida at species level is important because it helps guide appropriate treatment. HIV patients not on drugs should also be screened for oropharyngeal candidiasis because the presence of OPC in such individuals could be an indication to start anti-retroviral therapy.
